# Supporting Emerging Disciplines with e-Communities: Needs and Benefits

**DOI:** 10.2196/jmir.971

**Published:** 2008-06-30

**Authors:** Heiko Spallek, Brian S Butler, Titus K Schleyer, Patricia M Weiss, Xiaoqing Wang, Thankam P Thyvalikakath, Courtney L Hatala, Reza A Naderi

**Affiliations:** ^3^Health Sciences Library SystemUniversity of PittsburghPittsburghPAUSA; ^2^Joseph M Katz Graduate School of BusinessUniversity of PittsburghPittsburghPAUSA; ^1^Center for Dental InformaticsSchool of Dental MedicineUniversity of PittsburghPittsburghPAUSA

**Keywords:** Dental informatics, Internet, faculty, dental, education, dental, continuing, education, dental, graduate

## Abstract

**Background:**

Science has developed from a solitary pursuit into a team-based collaborative activity and, more recently, into a multidisciplinary research enterprise. The increasingly collaborative character of science, mandated by complex research questions and problems that require many competencies, requires that researchers lower the barriers to the creation of collaborative networks of experts, such as communities of practice (CoPs).

**Objectives:**

The aim was to assess the information needs of prospective members of a CoP in an emerging field, dental informatics, and to evaluate their expectations of an e-community in order to design a suitable electronic infrastructure.

**Methods:**

A Web-based survey instrument was designed and administered to 2768 members of the target audience. Benefit expectations were analyzed for their relationship to (1) the respondents’ willingness to participate in the CoP and (2) their involvement in funded research. Two raters coded the respondents’ answers regarding expected benefits using a 14-category coding scheme (Kappa = 0.834).

**Results:**

The 256 respondents (11.1% response rate) preferred electronic resources over traditional print material to satisfy their information needs. The most frequently expected benefits from participation in the CoP were general information (85% of respondents), peer networking (31.1%), and identification of potential collaborators and/or research opportunities (23.2%).

**Conclusions:**

The competitive social-information environment in which CoPs are embedded presents both threats to sustainability and opportunities for greater integration and impact. CoP planners seeking to support the development of emerging biomedical science disciplines should blend information resources, social search and filtering, and visibility mechanisms to provide a portfolio of social and information benefits. Assessing benefit expectations and alternatives provides useful information for CoP planners seeking to prioritize community infrastructure development and encourage participation.

## Introduction

### Science as a Collaborative Activity

Over the centuries, science has developed from a solitary pursuit into a team-based collaborative activity and, more recently, into a multidisciplinary research enterprise [1-3]. The increasingly collaborative character of science, mandated by complex research questions and problems that require many competencies, is evidenced by the creation of large research networks that share data or jointly use unique instruments. Barriers to such networks have been lowered by the advent of the Internet, which can provide an underlying electronic infrastructure for large collaborative efforts. Disciplines such as astronomy would not have developed as rapidly without joint construction and use of billion-dollar facilities; disciplines such as genomics cannot quickly advance without cross-correlating output data using a jointly developed sequence archive.

Biomedical research follows this trend closely, due in large part to federal funding initiatives such as the National Institutes of Health (NIH) Roadmap, which encourages the formation of multidisciplinary research teams as outlined in its “Research Teams of the Future” theme [4]. Recently, the NIH funded 12 institutions under its Clinical and Translational Science Awards (CTSA) program, which is designed to accelerate the transfer of results from basic science to clinical practice—an inherently multidisciplinary goal. Some of the awardees are trying to advance the science of doing science through collaboratively developed electronic applications, transforming their academic research centers into communities of science [5].

### Typology of e-Communities

However, the emergence of e-communities is not limited to multidisciplinary research teams but can be observed in many different contexts. E-communities have long been used to support collaboration among professionals and researchers [6-8]. More generally, e-communities are often created to facilitate interaction between people with similar needs, problems, or goals [9,10]. Considerable research has been devoted to characterizing these communities, making it possible to conceptually identify and describe pathways that can accelerate their emergence in the field of biomedical research.

**Figure 1:**
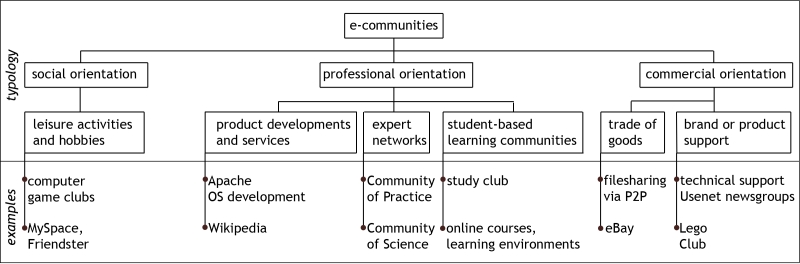
Characterization of e-communities (derived from [11])

E-communities can be characterized according to social, commercial, or professional orientation [11] (Figure 1). Social e-communities, such as MySpace [12], Friendster [13], and Facebook [14,15], evolve around leisure activities or hobbies. These communities originally consisted primarily of social software tools allowing members to meet new people. Commercial e-communities like eBay, which provides a platform for auctions among its worldwide community of 168 million members [16], focus on facilitating the marketing and selling of goods. Professional e-communities are formed around shared professional interests and can broadly be divided into entities focused on product development and services, expert-based knowledge networks, or student-based learning communities [17]. Examples of product- or service-based e-communities are the 75,000 contributors to the online encyclopedia Wikipedia [18] or the 2014 active developers [19] who work on Apache, an open source software product that has claimed 67% of the Web server market [20]. Expert-based knowledge networks, also referred to as communities of practice (CoPs), seek to expand, develop, and document existing knowledge by facilitating interaction between practitioners and researchers interested in a field.

### Communities of Practice

CoPs focus on one domain of knowledge and the accumulation of knowledge and expertise in this domain over time [21]. For instance, CoPs allow education professionals to support one another and enhance teaching [7,22]. An example of such an e-community is the Multimedia Educational Resource for Learning and Online Teaching (MERLOT) [23]. Organizational CoPs support efficiency and learning among knowledge workers [24]. According to Johnson, who distinguishes CoPs from traditional organizations, research communities have members with “different levels of expertise…simultaneously present,” allow for a “fluid peripheral to center movement that symbolizes the progression from being a novice to an expert,” and support “completely authentic tasks and communication” [25]. CoP participants receive new factual information, solutions to problems, and learning and insight [26]. Tapped In, for instance, allows isolated education professionals to support one another’s teaching efforts [7], and Math Forum promotes communication among researchers, practitioners, administrators, and students interested in the study and teaching of mathematics [22]. These benefits are derived from information that is socially embedded, existing in the context of interpersonal and group interaction, unlike the neutral authority-based information found in traditional sources such as journal literature [27].

Compared with the research performed on social and commercial e-communities and on professional e-communities focused on product development or services or on learning, research on CoPs lags behind. A thorough search for literature evaluating how well these systems facilitate the initiation of collaborations yielded no results. Judging from anecdotal evidence, systems of this type currently do not play a significant role in helping researchers establish collaborations. However, it is this type of e-community that is crucial for the transformation of biomedical research. Little is known about how socially embedded benefits can be exploited for the formation of CoPs. However, this is what programs like the CTSA aspire to, advancing science through communication among scientists from different fields with disparate primary research agendas. The research described in this paper focuses on the role e-communities can play in the genesis and growth of new or loosely formed fields or disciplines.

### Case Study: The Dental Informatics Online Community

The field examined in this case study is dental informatics (DI), which, unlike its parent discipline, biomedical informatics, can still be characterized as a nascent discipline [28]. Bridging different disciplines, DI is similar to other emerging disciplines such as pharmacogenetics and consumer health informatics. DI, which can be defined as the application of computer and information science to improve dental practice, research, education, and program administration [29], faces major challenges to establishing itself [28,30]. These challenges are similar to those of other emerging disciplines and include, for instance, a small, slowly growing number of geographically dispersed, experienced, trained researchers and the absence of a dedicated professional infrastructure such as a society or standing annual conference [31]. Therefore, DI seems to be an appropriate context for a study of how to overcome the characteristic challenges and hasten the development of emerging disciplines through collaborative electronic applications.

**Figure 2:**
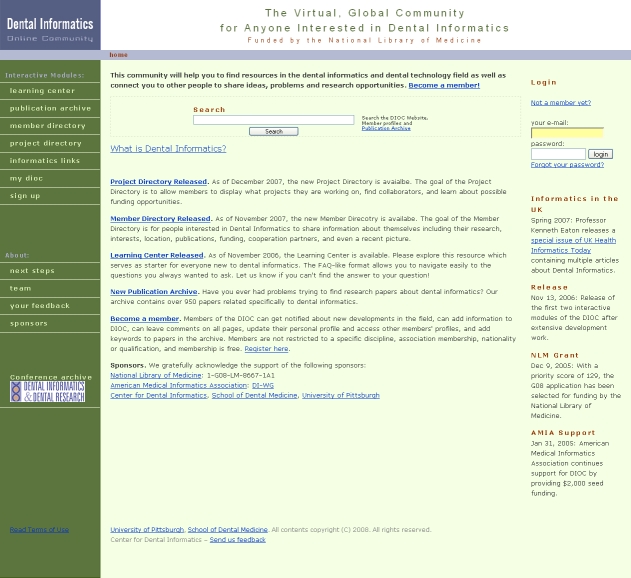
Screenshot of Dental Informatics Online Community (DIOC) home page

To these ends, a global e-community, the Dental Informatics Online Community (DIOC), is being established (Figure 2). Supported by an electronic infrastructure, the DIOC’s three project charges are as follows: (1) encouraging and supporting the formation of partnerships and collaborative projects in DI, (2) promoting the development of DI resources, and (3) disseminating research results and best practices. Ideally, the DIOC can provide a dedicated professional home for DI researchers and serve as an open, common, and worldwide forum for all individuals interested in the field.

### Challenges for New Communities of Practice

The DIOC, like any other new CoP, first needs to attract and retain a critical mass of participants by, for instance, widely advertising the expected benefits of participation. Unlike traditional information systems, a CoP depends on volunteers to provide content. Thus, after attracting participants, CoPs need to foster active participation. Studies of participation demographics in multi-user communities and social networks have found that between 46% and 82% of users are lurkers who never contribute [32]. While participation inequality cannot be entirely overcome, it must be recognized and addressed in order to achieve a reasonable diversity of contributing sources. On the other hand, legitimate peripheral participation should not be discouraged [33]. Even if the highly active core of members is the most crucial source of information, a viable e-community needs a steady flow of members with a range of commitment levels—peripheral and moderately engaged as well as highly active. CoPs generally strive not to encapsulate their members but instead to help them succeed outside the community. An external orientation is crucial because the DI research community has a responsibility to educate the wider dental community about DI’s scope and potential contributions.

The first step in attracting participants to a new CoP and then transforming many of them into active contributors is to determine the information needs of the target audience. There is general recognition that a needs assessment is the first step in any project that aims at providing useful information for a specific target audience [34-36]. While there is a large body of literature on the information needs of clinicians and health consumers, very few studies target the specific information needs of nonclinical biomedical researchers. The Faculty BurdenSurvey, evaluating the workload of university researchers, has shown that scientists spend 42% of their research time filling out forms and attending meetings. The results also reveal researchers’ struggles to find research partners and hire research personnel [37]. A distinguishing feature of researchers’ information needs is that they are not limited to bibliographical information or textbook facts, but also include knowledge about research infrastructure in such areas as funding, policy, and the training pipeline. Early studies show that scientific research is communal, reflecting a strong network of interconnected scientists who use formal and informal channels of information exchange [38-40].

This analysis leads to three main research questions:

1. Which information resources do researchers currently prefer to use?
2. How can their current professional relationships be described?
3. What are their expectations of a CoP, and how are these influenced by factors such as amount of participation necessary for a sustainable e-community and level of involvement in funded research?

The answers to these questions can assist with outlining the basic requirements for an e-community whose goal is to accelerate the emergence of a new discipline. While other successful e-communities could partially be used to model the DIOC, creating a community for a field in its formative stages requires more than just copying and pasting features and functions of e-communities for well-established disciplines. Thus, a needs assessment of prospective members was undertaken.

##  Methods

### Instrument

A review of the literature did not identify an existing instrument suitable for determining information needs and expected benefits. Thus, our first task was to develop such an instrument. Informal interviews with a convenience sample of four active DI researchers suggested some common information needs and revealed a strong desire for peer communication. Problems they identified with finding information sources as well as information needs identified in published studies were used as the starting point for an original survey instrument. These initial items were then developed and refined using Dillman’s Tailored Design Method [41] and principles from Thinking about Answers [42]. The survey design, delivery, and responses are reported here according to the Checklist for Reporting Results of Internet E-Surveys (CHERRIES) [43]. The final draft included 22 questions and was tested in a two-step process:

1. An expert group (three DI faculty [TKS, HS, TPT], three medical librarians [PMW plus two others], one business school faculty member [BSB], and one business school doctoral student [XW]) provided qualitative feedback. As a result of their evaluation, two questions were dropped, 12 were revised, and the texts of the preamble and email invitation were altered.
2. Nine volunteers from the target population participated in an evaluation using the Retrospective Thinkaloud protocol as suggested by Sudman at al [42]. This method avoids many of the pitfalls of concurrent narration such as disturbing the normal process of thinking about the answers. Volunteers received the survey ahead of time via email as an MS Word (Microsoft Corporation, Redmond, WA, USA) document with instructions not to open it before a 30-minute phone interview, during which we did the following:

- asked them to answer one survey question at a time
- engaged them in a short follow-up discussion after each answer
- inquired about the methods used to arrive at each answer
- logged their answers, problems, or comments
- solicited final comments and general suggestions

Evaluation of phone interview data resulted in further revision of seven of the 20 survey questions: in four cases, wording was not sufficiently comprehensive; in three, questions were too specific; in two, questions were misinterpreted. In addition, two more questions were eliminated, and two questions were combined into one.

The final version of the survey instrument included 17 items that were presented on one screen: five demographic questions, including current position; one question on expectations regarding the DIOC; six questions regarding professional relationships; and four questions about information-seeking behavior. There was also a general comments section at the end of the survey.

Three question formats were used. Two questions were open-ended, asking for extended text input; five questions were open-ended, with short answers such as age; and nine questions provided multiple-choice options. The question regarding participants’ expectations branched differently depending on whether or not they had already signed up for the DIOC; those who had signed up were also asked how they had learned about it (see the Multimedia Appendix).

The study was approved by the University of Pittsburgh’s Institutional Review Board in May 2006.

### Target Population

To increase the likelihood that the survey would provide representative data encompassing the needs of all people interested in DI, the composition of the prospective target audience was first determined. In addition to including clusters of people easily accessed through established gatherings such as the American Dental Education Association (ADEA) TechnoFair, an annual teaching technology showcase event of dental educators, we wanted to cover the possibility of unanticipated subgroups that might have their own membership or meeting organizations. To that end, we analyzed a set of 620 Medline abstracts identified for a 2003 study [31]. The combined approaches identified 12 distinct recruitment groups (Table 1).

**Table 1:** Distribution of target population across interest/source groups

Group Description^*^	Email Addresses, No.	Survey Respondents, No. (%)
Personally approached at AADR, ADEA 2006	113	28 (24.8)
Authors of 620 DI papers	910	58 (6.4)
AMIA DI working group member list	44	13 (29.5)
IMIA DI working group member list	133	24 (18.0)
Bioinformatics researchers with dental interest	11	3 (27.3)
ADEA TechnoFair authors (2004, 2005, 2006)	369	48 (13.0)
Current DIOC members	211	92 (43.6)
2003 DI conference participants	82	15 (18.3)
MLIS community	110	6 (5.6)
MLA (randomly selected 385 of the 3850-member directory)	385	6 (1.6)
280 funded informatics researchers (randomly selected 100)	100	1 (1.0)
9000 funded dental researchers (randomly selected 300)	300	14 (4.7)
		
Total	2768	–
Total after eliminating duplicates	2609	–
Total after eliminating duplicates and validating	2303	256^†^ (11.1)

^*^AADR, American Association for Dental Research; ADEA, American Dental Education Association; DI, dental informatics; AMIA, American Medical Informatics Association; DIOC, Dental Informatics Online Community; MLIS, Master of Library and Information Science; MLA, Medical Library Association.

^†^Total number of respondents is smaller than sum of group respondents because some individuals belong to more than one group.

Email addresses for individuals in the groups were obtained using two main approaches. Where member directories for organizations such as the American Medical Informatics Association (AMIA) DI working group were accessible, addresses were extracted directly from them. If member directories were not accessible, names and institutional affiliations were extracted from other publicly available sources; for example, current email addresses of the authors of 620 known DI papers from Medline [31] were obtained by manual search of their respective institutional websites. Duplicate email addresses within each group were eliminated.

At the time of the survey, the DIOC website had been operating and accepting registrations for 6 months. Although many DIOC features were not yet functional, 211 people had registered after finding the site either through publicity or their own search. These individuals were also invited to participate in the survey.

 All 2768 email addresses in the combined groups (see Table 1) were entered into a database (MySQL version 5.0.18; Sun Microsystems, Santa Clara, CA, USA). Duplicate entries were eliminated, but all information about group association was retained. In this intermediate collection, there were 2609 unique email addresses.

It was incidentally noted that after merging the 12 groups, there were only 158 duplicate addresses, indicating a very shallow overlap among target audience sectors. The majority of people who had not signed up for the DIOC (2354, 98.1%) belonged to just one of the sampled organizations; 40 were members of two organizations, and 5 belonged to three organizations. Among the 211 DIOC members, 136 (64.4%) did not belong to any other organization; 61 belonged to one other organization, 11 belonged to two others, and 3 belonged to three others. These observations are consistent with the common characterization of DI as a diverse but somewhat fragmented community. Overall, then, the sample seemed to include both a very small core of widely active participants and a large body of peripherally involved individuals.

In order to calculate a more accurate response rate, we tried to filter out nonexisting email addresses by programming an add-on to Sendmail (version 8.13.1; Sendmail Inc, Emeryville, CA, USA) and emailing the invitations from the server it ran on (Linux 2.6.9, Red Hat 3.4.5/Apache 2.2.0; Red Hat Inc, Raleigh, NC, USA; Apache Software Foundation, Forest Hill, MD, USA). The add-on program recorded and flagged 306 email addresses as nonexistent. After this process, 2303 unique email addresses remained. However, it was not possible to detect email accounts that, while technically operational, had been abandoned by users. As a result, the response rates reported here are biased low.

### Delivery Format

A Web-based format was chosen for the survey instrument because it significantly reduces turnaround time compared with mail surveys [44]. Because the goal is to establish an online community, concerns about Web-based surveys being biased toward computer users were not a significant issue [45]. All data were stored on a state-of-the-art administered server with LAMP architecture.

Invitations to complete the survey were emailed and included a unique access code to prevent both duplicate entries and completion by people who were not part of the target audience. Prospective participants were informed of how long the survey would take, who the investigators were, and that the data would be used for scholarly purposes only. Incomplete surveys could be submitted by respondents since no validation of user entries was performed. Thus, response rate for each question was different, as reported in the survey results below.

The initial invitation was emailed on June 1, 2006. A reminder was sent on June 14, 2006, and a final reminder was sent on July 10, 2006. No incentives were provided to any respondents.

### Data Analysis

After the survey closed on August 10, 2006, all response data in the MySQL database were exported to an MS Excel (Microsoft Corporation, Redmond, WA, USA) spreadsheet stored on a secure local file server. The majority of the survey questions required quantitative responses and could thus be analyzed with little or no additional manipulation. The open-ended questions regarding expected benefits of the CoP were coded into categories by two raters [BB, HS]. After agreeing on a 14-category coding scheme, both raters independently coded all individual responses. Disagreements on coding for specific items were resolved through discussion.

Analysis of the data included descriptive characterization of information-seeking and collaboration-related needs, examination of differing expectations within meaningful subsets, and identification of respondent clusters with distinctive expectations for a research-oriented online community. Comparison of the subsets was based on chi-square tests of difference in the relative proportions of the reported expectations. A two-step cluster analysis (implemented in SPSS version 15.0; SPSS Inc, Chicago, IL, USA) was used to determine the degree of homogeneity in benefit expectations. This exploratory procedure uses comparisons of individual responses (in this case, the benefits expected by each respondent) to identify sets of similar individuals. Examination of relative scores and *t* test results were then used to determine the specific benefits or benefit combinations that distinguished one cluster from the others.

## Results

### Response Rate and Demographics (Questions 12-17)

The response rate of 11.1% (256/2303) is based on the validated, unique email addresses. Of the 211 individuals already signed up as DIOC participants, 92 (44% of group and 36% of all respondents) completed the survey (see Table 1).

On average, respondents were 46.4 years old, had held their current title for 7.9 years, and had been at their current institution for 11.6 years. The 249 respondents to the question on country of residence reported living in 30 different countries (Table 2). A plurality held academic positions of varying rank; many of the others identified themselves as students, dental practitioners, or scientists (Table 3). To assess the representativeness of the respondents, we compared their main professional activity with our initial target group association using Pearson correlation. We found no significant correlation between the respondents’ main professional activity and their initial target group association (*P* < .05).

**Table 2:** Distribution of respondents’ country of residence (partial list, only countries mentioned at least three times)

Country	No. (%)
United States	139 (54.3)
Germany	15 (5.9)
Canada	10 (3.9)
United Kingdom	7 (2.7)
Netherlands	7 (2.7)
India	6 (2.3)
Australia	4 (1.6)
Sweden	4 (1.6)
Italy	4 (1.6)
Japan	3 (1.2)
Missing responses	7 (2.7)
	
Total number of respondents	249 (97.3)
Total	256 (100)

**Table 3:** Distribution of respondents’ academic positions (partial list, only positions mentioned at least twice)

Academic Position	No. (%)
Full professor	36 (14.1)
Associate professor	35 (13.7)
Department chair/CEO/director	25 (9.8)
Postgraduate student	21 (8.2)
Dental practitioner	18 (7.0)
Scientist	17 (6.6)
Consultant	13 (5.1)
Administrator	11 (4.3)
Librarian	7 (2.7)
Dean	6 (2.3)
Predoctoral student	3 (1.2)
Dental hygienist	2 (0.8)
Missing responses	25 (9.8)
	
Total number of respondents	231 (90.2)
Total	256 (100)

### Information-Seeking Behavior (Questions 1, 2, 5, 6)

Table 4 shows that electronic resources dominate as information sources for the target audience when asked, “How often do you use the following information sources when trying to find professional information?”

**Table 4:** Use of information sources*

Information Source	Frequently, No. (%)	Sometimes, No. (%)	Never, No. (%)	Total
Medline (via Ovid, PubMed, EMBASE, Web of Knowledge, or other database provider)	196 (80.3)	35 (14.3)	13 (5.3)	244
Internet search engines (Google, Yahoo, Lycos, etc)	186 (83.4)	35 (15.7)	2 (0.9)	223
Online journals (e-print, full-text archives of print journals, etc)	184 (78.6)	48 (20.5)	2 (0.9)	234
Print journals	114 (47.5)	117 (48.8)	9 (3.8)	240
Books from your personal collection	103 (44.4)	113 (48.7)	16 (6.9)	232
Conferences, lectures, etc	94 (40.7)	134 (58.0)	3 (1.3)	231
Researchers within my institution	89 (38.7)	115 (50.0)	26 (11.3)	230
Researchers from other institutions	70 (30.7)	143 (62.7)	15 (6.6)	228
Books from/in libraries	61 (26.3)	137 (59.1)	34 (14.7)	232
Bibliographic databases such as… Cochrane Database of Systematic Reviews or other Cochrane Library components	61 (26.5)	93 (40.4)	76 (33.0)	230
Newsletters	60 (26.0)	127 (55.0)	44 (19.0)	231
National or local media (newspapers, television, etc)	51 (22.0)	114 (49.1)	67 (28.9)	232
Other information source: which?	48 (60)	32 (40)	N/A	80
IEEE Xplore	20 (9.4)	41 (19.3)	151 (71.2)	212

^*^Responses to the following question: “How often do you use the following information sources when trying to find professional information?”

Asked about the existence and use of an institutional library, 213/251 respondents (84.9%) indicated that they have access to one, and 194 (91.1% of those indicating access) do use it either physically or virtually.

There were 162 responses to an open-ended question regarding the manner in which the respondents find out about research funding. Funding resources were identified mostly through visits to known funding agencies’ websites, frequently those of NIH. Next in frequency were various forms of intra-institutional notification; personal communication, including not only formal contact but also informal word of mouth; and use of general Web search engines. Among the 16 resources that were categorized as aggregating services, Community of Science was mentioned most often.

### Professional Relationships (Questions 3, 4, 7-9)

Respondents were asked about collaboration, with collaborator defined as “co-author, co-investigator, consultant to a specific project” (Question 3). During the previous 12 months, 193 respondents had, on average, worked with 10 collaborators. Table 5 summarizes collaborator origins.

**Table 5:** Origin of collaborators during the past 12 months (multiple selections were permitted)

Options for Origin of Past Collaborators	No. (%)
Come from my department	179 (92.7)
Come from other institutions with faculty specializing in my area of interest	173 (89.6)
Come from my institution, outside my department	172 (89.1)
Are people with whom I have collaborated in the past	170 (88.1)
Are people with whom I have conducted relevant research	133 (68.9)
Are people whom I met at conferences, conventions, etc	119 (61.7)
Are people to whom I was introduced to by a colleague	111 (57.5)
Other	38 (19.7)

When asked where they usually find research assistants (Question 4), most of the 248 respondents reported getting help from inside their institution (mentioned 86 times, 34.7%), from past helpers (mentioned 74 times, 29.8%), or from inside their department (mentioned 69 times, 27.8%) rather than from recruitment services within (mentioned 43 times, 17.3%) or outside (mentioned 26 times, 10.5%) their organization.

On average, respondents attend five professional meetings per year (based on 245 respondents to Question 7). Relevance of the meeting agenda to one’s general research interests, relevance to particular research projects, and potential for networking with fellow researchers were the crucial criteria used in deciding meeting attendance (Table 6). Less important were whether the conference featured an esteemed researcher and the availability of funding to support attendance.

**Table 6:** Factors influencing conference attendance*

Factor	Very Important, No. (%)	Somewhat Important, No. (%)	Not Important, No. (%)
Relevance of agenda to my general research interests	168 (65.6)	51 (19.9)	7 (2.7)
Relevance of agenda to a particular research project	122 (47.7)	85 (33.2)	14 (5.5)
Conference features an esteemed researcher	48 (18.8)	121 (47.3)	49 (19.1)
Likelihood of attendees’ research interests coinciding with my own	82 (32.0)	108 (42.2)	31 (12.1)
Networking with fellow researchers	109 (42.6)	90 (35.2)	21 (8.2)
Availability of funding to support attendance	88 (34.4)	73 (28.5)	60 (23.4)
Ability to present my own work	92 (35.9)	91 (35.5)	37 (14.5)
Other	34 (13.3)		
Missing responses	7 (2.7)		
	
Total number of respondents	249 (97.3)		
Total	256 (100)		

^*^Responses to the following question: “To what degree do the following factors influence whether you attend a particular conference or not? (Rate the factors.)”

Respondents were asked if they belonged to specific dental and informatics organizations (Question 9). They could augment their response by entering up to three additional organizations; 130 respondents (56.3%) belonged to the International Association for Dental Research (IADR), 97 (42.0%) to the American Dental Education Association (ADEA), and 77 (33.3%) to the American Dental Association (ADA). A total of 88 respondents were members of one of the listed organizations, 59 of two organizations, and 30 of three organizations. The most common write-in choices were European dental research and medical specialty organizations.

### Expectations for the DIOC (Questions 10, 11)

**Figure 3:**
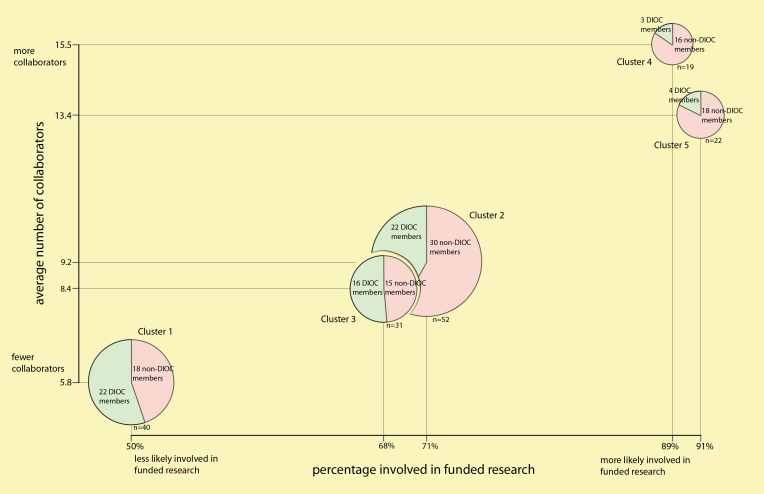
Benefit clusters

Participants who had already signed up for the DIOC were asked about what kinds of benefits they expected from their involvement. Those who had not signed up were asked how they thought an e-community might help them with their research; 64% (164/256 respondents, both groups combined) reported at least one type of expected benefit. The two raters coded the individual responses on a 14-category coding scheme (Kappa = 0.834), concentrating on how benefit expectations related to (1) the respondents’ willingness to participate in the DIOC and (2) how this willingness was related to involvement in funded research (Figure 3).

#### DIOC Versus Non-DIOC Participants

Individuals who had already signed up for the DIOC tended to expect more specific benefits from the community than those who were not yet registered, including general information, identification of experts, networking with peers, advocacy support, and career development (Table 7). However, there may have been confounding factors such as the different question construct (see Multimedia Appendix for the survey instrument) and the fact that DIOC members were primed by reading the goals of the community when they initially signed up.

**Table 7:** Comparison of expected benefits mentioned by different groups

Benefit Category	DIOC Membership			Research Funding			Total, No. (%), (n = 164)
	Non-Member, No. (%), (n = 97)	Member, No. (%), (n = 67)	P^*^	Funded, No. (%), (n = 115)	Not Funded, No. (%), (n = 49)	P^*^	
Information Benefits	72	63		93	42		135
General information	38 (39.2)	47 (70.1)	< .001	56 (48.7)	29 (59.2)	.15	85 (51.9)
Funding information	18 (18.6)	4 (6.0)	.02	20 (17.4)	2 (4.1)	.02	22 (13.4)
Specific topic	10 (10.3)	7 (10.4)	.59	12 (10.4)	5 (10.2)	.60	17 (10.4)
Teaching materials	4 (4.1)	2 (3.0)	.53	3 (2.6)	3 (6.1)	.25	6 (3.7)
Data sharing	2 (2.1)	3 (4.5)	.33	2 (1.7)	3 (6.1)	.16	5 (3.1)
							
Social Benefits	45	69		74	40		114
Peer networking	21 (21.6)	30 (44.8)	.00	35 (30.4)	16 (32.6)	.46	51 (31.1)
Identification of potential collaborators and/or research opportunities	19 (19.6)	19 (28.4)	.13	30 (26.1)	8 (16.3)	.12	38 (23.2)
Advocacy support	2 (2.1)	9 (13.4)	.01	5 (4.3)	6 (12.2)	.07	11 (6.7)
Expert identification	1 (1.0)	6 (9.0)	.02	2 (1.7)	5 (10.2)	.03	7 (4.3)
Participation in the field	2 (2.1)	5 (7.5)	.10	2 (1.7)	5 (10.2)	.03	7 (4.3)
							
Instrumental Benefits	3	8		7	4		11
Career development	1 (1.0)	7 (10.4)	.01	4 (3.5)	4 (8.2)	.19	8 (4.9)
Recruiting	2 (2.1)	1 (1.5)	.64	3 (2.6)	0 (0.0)	.34	3 (1.8)
							
Other Benefits	23	11		26	8		34
Uncertain	17 (17.5)	3 (4.5)	.01	18 (15.6)	2 (4.1)	.03	20 (12.2)
Unclassifiable	6 (6.2)	8 (11.9)	.16	8 (7.0)	6 (12.2)	.21	14 (8.5)
							
Average number of benefits cited per respondent	0.87	1.64		1.24	0.99		

^*^Determined by chi-square analysis.

#### Funded Versus Not Funded Research

The approximately 70% of respondents who participate in funded research were significantly more likely to expect the DIOC to be a source of funding information and opportunities (see Table 7). Number of collaborators, an indicator of research involvement, was positively correlated with the expectation that the DIOC might provide information about funding opportunities (Spearman correlation = 0.164, *P* = .049) and recruiting (Spearman correlation = 0.184,*P* = .03). By contrast, individuals not participating in funded research were more likely to expect the DIOC to help them with expert identification and opportunities to participate in the field.

Active researchers were significantly more likely than non-researchers to express uncertainty concerning the potential benefits of participation in the DIOC. Number of collaborators was also positively correlated with the likelihood of a respondent reporting uncertainty (Spearman correlation = 0.229, *P* = .01). Tenure in current position was negatively correlated with expectations of receiving general information benefits (Spearman correlation = −0.18, *P* = .03).

### Benefit Clusters

Overall, the most frequently expected benefits from participation in the DIOC were general information (eg, exchange of ideas, keeping well informed), mentioned by 51.9% of respondents; peer networking (eg, finding colleagues with same interests), mentioned by 31.1%; and identification of potential collaborators and/or research opportunities, mentioned by 23.2%. Two-stage cluster analysis revealed five identifiable clusters, each associated with a distinctive collection of benefit expectations (see Figure 3):

- Cluster 1: General information
- Cluster 2: General information and social benefits (collaboration, peer networking, etc)
- Cluster 3: General information and peer networking
- Cluster 4: Uncertainty
- Cluster 5: General information and collaboration opportunities

General information benefits were widely mentioned across all clusters, but responses regarding social benefits varied. While 58% mentioned some type of social benefit, the cluster analyses suggest that some individuals seek general information alone, while others expect general information combined with peer networking and collaboration opportunities.

In addition to reflecting specific combinations of benefits, the clusters were also distinguished by the characteristics of the individuals associated with them. Individuals in Clusters 1 and 2 tended to have fewer collaborators, be less likely to be doing funded research or using online search resources (Medline, Cochrane Library), and be more likely to have signed up for the DIOC. By contrast, members of Cluster 4 were proportionately more likely to be participating in funded research and to have a higher number of collaborators. Members of Cluster 5 were more likely to have a higher number of collaborators, more likely to be doing funded research, and less likely to have signed up for the DIOC.

### Knowledge About the DIOC

Of the individuals who had already signed up for the DIOC, 36/91 respondents (40%) learned of it via an Internet search engine, 26 (29%) received an electronic announcement, 19 (21%) heard about it during a conference, and 22 (24%) specified other sources. Respondents were allowed to select multiple responses for this question.

## Discussion

### Principal Findings

Any online community must attract a critical mass of involved participants if it is to be sustainable. Individual researchers develop expectations about the benefits of involvement, and these benefit expectations play a significant role in their satisfaction with, commitment to, and, ultimately, participation in an e-community [46]. While information needs continue to figure prominently in expectations, researchers are increasingly seeking support for the social aspects of information use and tools that support formation of collaborative relationships. Understanding benefit expectations (both on their own and in the larger socio-informational context) and developing technical infrastructure and resources to meet them are critical to facilitating biomedical research with CoPs.

### Information Needs and Implications for Community Design

Up-to-date information resources are a foundational element of any planned CoP. Access to a variety of timely information was often mentioned as a desirable benefit of involvement in the DIOC by individuals across all clusters. The DIOC’s planned information stores, including general information about DI as well as more specific resources such as a project directory, address this need.

The ideal is for community participants to generate a significant proportion of information resources themselves in such forms as detailed personal profiles, postings to the project directory, and tags, comments, and other annotations. But it may be difficult to quickly attain and then sustain such a goal to a degree that satisfies researchers accustomed to immediate access to plentiful and readily available traditional library resources—not to mention the abundant, if unvetted, resources of the Web. In addition, a CoP needs to offer an attractive breadth and depth of material without creating an undue content creation burden on each participant. Thus, DIOC planners may need to allocate ongoing funds for creation and maintenance of information resources to augment content created by participants, such as a mix of searchable databases and interactive features that can accommodate the anticipated range of user expectations and behavior. Whether this challenge exists for research-oriented CoPs in general is a question for future research.

Just as respondents judge the value of a conference or meeting by how well its topic matches their interests or has particular relevance to a specific research project, potential CoP participants see information resources as an indication of the fit between community activities and their own needs and interests. However, since any one individual is likely to be interested in only a fraction of the available material, CoP architectures and interfaces must include targeting and filtering capabilities. For example, CoPs should aggregate timely information about funding opportunities relevant to their prospective audiences and automatically alert users to new funding opportunities in a targeted manner. These notifications need to match user subject interests and accommodate user preferences [47-50].

### Social Information Use and Implications for Community Design

The high degree of reliance on personal communications and word of mouth (mentioned 34 times out of 162 responses) indicates that even with electronic alerts and Internet searches, personal communication remains a significant source of information about funding opportunities for our respondents. This finding matches the results of earlier studies regarding the information-seeking behavior of dentists [51].

To support social information seeking and sharing, CoPs need infrastructure for both direct communication (such as document sharing and referrals) and indirect information sharing (via collective tagging or public annotation of informational items). CoPs also should provide contexts such as message boards and forums in which individuals who lack well-developed interpersonal networks can observe and participate in group discussion. Allowing CoP members to annotate, comment on, and discuss information will not only add value to the CoP, but will also encourage the building of trust and knowledge in the community, which are important elements in the development of computer-mediated interaction [52-54].

### Collaborative Relationship Needs and Implications for Community Design

Discipline- and research-oriented CoPs need to support professional relationships among members, enabling individuals to find potential collaboration partners and to form and maintain relationships. Our respondents’ collaborations originated almost equally from inside and outside their own departments and institutions, substantiating the findings of Griffith and Miller [39]. The global character of the DIOC makes it a potential site for forming collaborations outside members’ local institutions.

One key aspect of relationship formation is visibility. Increasing the visibility of individuals, their interests, and their intentions helps catalyze effective professional relationships. Each CoP member should be able to create and maintain a profile accessible to all, enabling subscribers to construct and develop verifiable identities within the community [52]. Profiles should not only include interests, location, collaboration partners, and publications, but should also point to information contributed to the CoP as a trace of the subscriber’s activity. A project directory and a research opportunity exchange will help members learn about each others’ current activities, find help for their own projects, and join projects in early stages as collaborators.

In addition to forming collaborative relationships with other individuals or other participant subgroups, individuals also want to develop and maintain awareness of what the overall community is doing. Emerging disciplines usually do not support a standing professional meeting, but CoPs can provide at least a partial substitute for that aspect of scholarly activity and for the networking opportunities generally available at traditional professional meetings. As mentioned above, it is hoped that the DIOC will substitute for a standing DI conference and serve as a professional home for researchers who primarily dedicate their career to this emerging discipline, allowing virtual affiliation without travel. Again, closely linked project and people directories that let members learn about ongoing projects and who is responsible for them are key resources.

### Online Communities as Part of a Complex Socio-Informational Ecology

Respondents with higher numbers of collaborators and involvement in funded research were more likely to express uncertainty about the benefits of participation. They were more likely to mention general information and collaboration opportunities as expectations, while those with fewer collaborators and no funded research participation mentioned social benefits such as expert identification and advocacy support. These differing profiles, coupled with the significant negative correlation between tenure in an organization and the expectation of general information benefits, underscore the fact that academic online communities such as the DIOC are competing with individuals’ own environments—their networks, institutions, and other immediately available resources.

Unlike traditional information systems, which are typically seen as the only, or at least the primary, source of information of a particular type within an organization, CoPs operate within a much broader, highly competitive social-information ecology. CoPs compete with individuals’ own local resources, so persuading time-pressured researchers to move from habitual exclusive reliance on known resources to exploring new tools and techniques in the interest of improving long-term productivity is a key challenge [5].

Individuals uncertain about benefits were proportionately more common among those who had not signed up for the DIOC (*P* < .05), highlighting the need to clearly demonstrate the benefits of participation during the recruitment process. In general, CoP planners faced with competition and potential users’ ambiguity need to consider the benefit stream visible to individuals approaching the community for the first time. They should provide an immediate payoff and participation incentives for first-time members of all types, with collaboration opportunities for those wanting a social context and straightforward information benefits for those whose expectation of social benefits is lower.

However, in complex ecological systems, attempting to “win” simply by direct competition can be a costly approach that often fails. The CoP planner should look for ways in which the presence of related resources and systems supports the goals of the community. For example, the use of online information sources by DI researchers, the emergence of the Internet as an important tool for dentists [55], and the advent of Google as an important clinical information resource for physicians [56] can be seen as either a competitive threat or an opportunity. Individuals’ reliance on online searching creates several positive externalities that CoP planners can take advantage of. CoPs can use state-of-the-art user interface design and search technology that is already familiar to the target group (eg, similar to those of Google or PubMed). Application programming interfaces and other affordances already provided by such applications can facilitate integration into the presentation of CoP resources. Lastly, the presence of a developing ecology of information sources and social computing tools allows CoP planners to incorporate resources and capabilities into a community without bearing the full cost of development and maintenance.

Taken together, these results characterize both the promise and the challenge of academic online communities. On the one hand, CoPs present clear benefits for individuals who are more isolated, less connected, and lacking in access to local institutional resources; these participants can, in return, increase the diversity and impact of an otherwise fragmented discipline such as DI [57,58]. On the other hand, they do require contribution of resources by their members if they are to provide substantial ongoing benefits [59]. Contribution in the form of participation creates a stronger and more valuable community. But ability and willingness to contribute are, in part, dependent on one’s local environment [60]. Thus, academic e-communities such as the DIOC face a paradox: the individuals best qualified to contribute to them are the least likely to see them as providing resources or benefits beyond those already available in their own professional milieus.

Yet the structure of the clusters in the DI community suggests a possible solution. By building a base of commonly valued information resources and providing individuals with the ability to pick and choose the nature of their social engagement with the community, the DIOC can provide an infrastructure that brings together a diverse group of individuals with complementary needs. Identifying the interlocking contribution-benefit pairs allows them to be addressed, and leveraged, during implementation [61]. Frameworks and strategies for identifying and working with complementary pairing of contributions and benefits should be pursued in future studies of CoPs.

### Limitations

A response rate of 11.1% is low but within the expected outcome range [62] given the fact that many of the email addresses used came from sources of unknown update status such as academic department home pages. The validation process eliminated some but not all of the invalid addresses. Thus, the reported response rate, while more accurate than it would have been without address validation, is likely to be an underestimate of the true response rate. Because a part of the results focuses on DIOC members, one needs to consider the influence of our earlier announcements as well as the material provided to the members on the preliminary website during sign-up. The specific language used in the marketing—“get involved…communicate with peers…disseminate research results…formation of research and education partnerships”—may have skewed baseline expectations.

Data about current position and country of residence show that respondents were well distributed across the spectrum of the intended target audience. The results seem to reflect the fact that interest in DI is spread among many different countries and pursued by people in various academic and clinical positions. However, it is possible that the selection of 12 target audience groups might not be entirely inclusive.

Some of the general comments made on the concluding survey question (“Is there anything else you’d like to tell us?”) criticized our US-centric view. While it is true that most of the professional organizations listed as choices for membership were US-based, the results of our pilot tests did indicate predominance of US respondents. However, a pro-US bias might have influenced question constructs and results.

This study relied on self-reported data, which may be incomplete and/or incorrect. For instance, respondents might have unperceived information needs that they did not report [35].

### Conclusions

We were able to assess the information needs of dental informaticians, researchers, educators, clinicians, and other interested parties. Data on expected benefits of a CoP for DI were collected and evaluated, allowing compilation of requirements for the creation of the DIOC.

The survey itself has increased the awareness of the DIOC project. Casual observation has shown that DIOC registration spiked in the wake of the various survey invitations and reminders.

Future work should focus on validating the instrument used in this study as well as carefully applying our findings to other emerging biomedical research fields such as consumer health informatics.

## Multimedia Appendix

Survey instrument, including branching differences

## Abbreviations

CoP: community of practice
CTSA: Clinical and Translational Science Awards
DI: dental informatics
DIOC: Dental Informatics Online Community
NIH: National Institutes of Health
