# Supplementary material for: Supporting Emerging Disciplines with e-Communities: Needs and Benefits
Source: J Med Internet Res. 2008 Jun 30;10(2):e19. doi: 10.2196/jmir.971 (PMC2483921; doi:10.2196/jmir.971)
Supplement: Supplementary file 1 [file jmir_v10i2e19_app1.pdf]

J Med Internet Res 2008;10(2):e19 (http://www.jmir.org/2008/2/e19)

Supporting Emerging Disciplines with e-Communities: Needs and Benefits

Multimedia Appendix 1: Survey instrument

How can a dental informatics online community help you?

The survey is anonymous. All responses will be treated as confidential data and reported only in summary form. Your participation is voluntary, and you may leave the survey website at any time.

This study is being conducted by Heiko Spallek, DMD, PhD, who can be reached at (412) 648-8886 or hspallek@pitt.edu. The ethics committee (Institutional Review Board) at the University of Pittsburgh has reviewed and approved this research protocol (IRB# 0604033).

Thanks for your time and effort! Your input will help us develop the Dental Informatics Online Community into a useful resource for your research efforts.

Sincerely,

Heiko Spallek, DMD, Ph.D.: hspallek@pitt.edu
Asst. Professor, Center for Dental Informatics
School of Dental Medicine, University of Pittsburgh
3501 Terrace Street, Pittsburgh, PA 15261

1. How often do you use the following information sources when trying to find professional information?

**Source**	**Frequently Use**	**Sometimes Use**	**Never Use**
online journals (e-print, full-text archives of print journals, etc.)	❍	❍	❍
hardcopy journals	❍	❍	❍
newsletters	❍	❍	❍
books from/in libraries	❍	❍	❍
books from your personal collection	❍	❍	❍
national or local media (newspapers, television, etc.)	❍	❍	❍
conferences, lectures, etc.	❍	❍	❍
researchers **within** my institution	❍	❍	❍
researchers **from other** institutions	❍	❍	❍
Internet search engines (Google, Yahoo, Lycos, etc.)	❍	❍	❍
Bibliographic databases such as… Cochrane Database of Systematic Reviews or other Cochrane Library components	❍	❍	❍

MEDLINE (via Ovid, PubMed, EMBASE, Web of Knowledge, or other database provider)	❍	❍	❍
IEEEXplore	❍	❍	❍
Other information source: which?			

2. Do you have access to an institutional library? ❍Yes / ❍No
If yes, do you use it (physically or via electronic means)? ❍Yes / ❍No

3. During the past 12 months, you have been collaborating with
_____number of collaborators (collaborator = co-author, co-investigator, consultant to a specific project)

Your collaborators … (Check as many as apply.)
❑ come from my department
❑ come from my institution, outside my department
❑ come from other institutions with faculty specializing in my area of interest
❑ are people with whom I have collaborated in the past
❑ are people whom I met at conferences, conventions, etc.
❑ are people with whom I have conducted relevant research
❑ are people to whom I was introduced to by a colleague
other: ____

4. How do you usually find research assistants, such as student workers, graduate assistants, full-time research associates (Check as many as apply.)?
If you do not require research assistants, check here ❑ and skip to the next question.

Location-based recruiting within my
❑ department
❑ institution, but outside my department

Approaching individuals directly
❑ who have assisted me with my past research projects
❑ whom I have met at conferences, conventions, etc.

Formalized recruiting methods using
❑ a recruiting, employment, or matching service, within my institution
❑ a recruiting, employment, or matching service, independent of my institution

other: ____

5. How do you find out about funding opportunities (describe briefly)?
____
____
____

❑I do not participate in funded research (Skip to Question 7).

6. How often do you use the following sources to identify funding opportunities?

Source	Frequently Use	Occasionally Use	Never Use
colleagues	❍	❍	❍
websites of prospective funding sources	❍	❍	❍
websites **specific to your field** or area of interest	❍	❍	❍
**general** research support websites	❍	❍	❍
NIH website/newsletter	❍	❍	❍
NSF website/newsletter	❍	❍	❍
IEEEXplore	❍	❍	❍
your own institution's research office website or its postings or mailings	❍	❍	❍
Other: which?	❍	❍	❍

❑ This does not apply to me; I do not participate in funded research.

7. How many conferences, conventions, or other professional meetings do you attend each year?
_____ / year

8. To what degree do the following factors influence whether you attend a particular conference or not? (Rate the factors)

Factor	Very Important	Somewhat Important	Not Important
relevance of agenda to my general research interests	❍	❍	❍
relevance of agenda to a particular research project	❍	❍	❍
conference features an esteemed researcher	❍	❍	❍
likelihood of attendees' research interests coinciding with my own	❍	❍	❍
networking with fellow researchers	❍	❍	❍
availability of funding to support attendance	❍	❍	❍
ability to present my own work	❍	❍	❍
other: which?	❍	❍	❍

9. Please list the most important professional associations or academic organizations that you are involved with.
❑ IADR, International Association for Dental Research

❑ ADA, American Dental Association
❑ AMIA, American Medical Informatics Association
❑ IEEE, The Institute of Electrical and Electronics Engineers
❑ ADEA, American Dental Education Association
❑ MLA, Medical Library Association
❑ AACR, American Association for Cancer Research
❑ HIMSS, Healthcare Information Management Systems Society
other: ______________________________
other: ______________________________
other: ______________________________

Begin Branching
Existing members

10. How did you hear about Dental Informatics Online Community (DIOC)?
❑ received a print announcement
❑ received an electronic announcement
❑ during a conference
❑ from colleague
❑ via an Internet Search
❑ other:______________________________

11. What are the three main benefits you expect from being involved with the Dental Informatics Online Community?
1. ______________________
2. ______________________
3. ______________________

Non-members

10. How could an online community for dental informatics (=a Website that provides information resources and allows you to interact with others) help you in your research?

End Branching

12. Which best describes you?
My main professional activity:

Informatics-focused
❍ biomedical informatics
❍ dental informatics

Dentistry-focused
❍ clinical dentistry
❍ dental/craniofacial research
❍ computer/information science
❍ cognitive science

Technology-focused
❍ dental information technology industry
❍ engineering
❍ telecommunication

Education-focused
❍ dental education
❍ medical education

Other
❍ library and information sciences
❍ administration

13. Please specify your title or your position (for instance, assistant professor or postgraduate student):

____

How long have you held this title? _____ years

14. How long have you been at your current organization or institution?
_____ years

15. Country of residence: [Drop-down Menu]

16. Your Age: _________

17. Your gender: ❍ male ❍ female

Is there anything else you'd like to tell us?
____
____
____

Send my response!

University of Pittsburgh School of Dental Medicine. All contents copyright (C) 2006. All rights reserved.
Last Update: May 31, 2006
Center for Dental Informatics - Comments to author: cdi@pitt.edu
